# Identifying performance differences between two pulse oximetry systems in simulated critical neonatal conditions

**DOI:** 10.1038/s41372-025-02364-4

**Published:** 2025-07-30

**Authors:** Brian King, Jake Dove, Scott J. McGonigle, Warwick A. Ames, Zachary A. Vesoulis

**Affiliations:** 1 StarFish Medical, Victoria, BC Canada; 2 Research and Development, Acute Care Monitoring, Medtronic, Boulder, CO USA; 3 Research and Development, Acute Care Monitoring, Medtronic, Midlothian, UK; 4 Duke University Medical Center, Durham, NC USA; 5 Department of Pediatrics, Washington University School of Medicine, St. Louis, MO USA

**Keywords:** Translational research, Circulation

## Abstract

**Objective:**

Pulse oximetry is used to guide critical clinical decisions in neonatology. We used a vital signs simulator to compare performance of two pulse oximetry systems in conditions not tested in standardized clinical verification studies.

**Study design:**

We devised a set of simulated tissue translucency, perfusion, peripheral oxygen saturation (SpO_2_)_,_ and heart rate (HR) parameters to mimic challenging real-world neonatal data and applied them to two marketed pulse oximetry systems (Nellcor™ and Masimo®). At each combination of input parameters, we used the response from both systems to assess SpO_2_ error.

**Results:**

The mean SpO_2_ error for Nellcor™ was below 1.1% across all parameters explored, while Masimo® showed significantly higher (*p* < 0.005) error at lower translucencies.

**Conclusion:**

Significant performance differences can be observed when comparing pulse oximeters at low translucency and perfusion conditions. Patient simulators cannot replace clinical testing but provide a safe and cost-effective method for additional performance profiling.

## Introduction

Pulse oximetry has become a standard of care for continuous monitoring in the neonatal intensive care unit (NICU), and is used in a variety of contexts, including guiding resuscitation in the delivery room [1, 2], monitoring of oxygen saturation in the operating room, and more recently, to screen for critical congenital heart disease (CCHD) [3–5].

Further, pulse oximetry is used to guide careful titration of respiratory support in the first few days of life, when preterm neonates typically undergo a transition in physiology and an accurate evaluation of subclinical hypoxemia is critical for the evolving diagnostic and medical management. Preterm infants must maintain a tenuous balance when receiving supplemental oxygen, as small changes in the level of oxygenation have been associated with adverse outcomes such as retinopathy of prematurity, intraventricular hemorrhage and increased mortality [6–11].

Despite being widely used in neonatal clinical practice, there are specific characteristics of the neonate which can challenge the accuracy of pulse oximetry systems, including low tissue translucency, poor perfusion and motion artifacts [1, 9–12]. Low translucency conditions are mainly due to dark skin pigmentation or thick tissue sites (pulse oximetry sensors are typically positioned on the neonates’ hands or feet, as their digits are simply too small). Moreover, neonates, especially those born preterm, have proportionally lower blood pressures, including a pulse pressure only about 50% of the magnitude of adults [13]. These infants also have immature peripheral vascular autoregulation, with diminished distal blood flow, further complicated by thermoregulatory instability [14]. The net effect of these factors is a marked reduction in the signal to noise ratio in pulse oximeters readings, which is difficult to replicate in healthy adult subjects on whom these devices are traditionally clinically verified.

Current methods for accuracy verification of a pulse oximeter used on neonates, outlined by the Food and Drug Administration (FDA) guidance [15] and by the International Organization for Standardization (ISO) 80601-2-61 standard [16], involve a controlled hypoxia study—limited to healthy adults for ethical and practical reasons—coupled with a convenience sampling study on neonates with arterial lines already in place for verification of clinical performance. These standardized methods are necessary to confirm the calibration curve for a pulse oximetry system [17], which is key for an accurate conversion from optical signals to peripheral oxygen saturation (SpO_2_) readings. However, they do not challenge the pulse oximetry systems over the entire space of possible neonatal signals; specifically, they do not assess accuracy of these systems in the above-mentioned low translucency and low perfusion conditions at varying saturation levels.

Patient simulators, designed to create synthetic signals that mimic human vital signs and equipped with extensive configuration options (including varying simulated vital parameters and various signal artifacts), may fill the gap between controlled standardized studies and uncontrolled real-world clinical settings without posing additional risks to fragile subjects. Simulators cannot replace standardized clinical testing, as they are unable to fully reproduce the actual tissue-sensor interface [17], which is essential to assess the clinical accuracy of pulse oximetry systems. However, simulators can be used to challenge and improve the design of these systems and may provide a streamlined, cost-effective method for supplemental pre-clinical verification of their performance [18].

In this work, a cohort of real-world pulse oximetry data gathered from the NICU was used to inform a bench test comparison of SpO_2_ monitoring performance of two widely used pulse oximetry systems in low-translucency and low-perfusion conditions representative of neonatal patients.

## Methods

### Pulse oximetry systems under test

This work compared the performance of the following two pulse oximetry systems:

1. Nellcor™ system: a Nellcor™ OxiMax™ N-600x patient monitor (Medtronic, Minneapolis, MN) coupled with a set of seven distinct DS100A-1 finger sensors.
2. Masimo® system: a Rad-97™ Pulse CO-Oximeter® (Masimo®, Irvine, CA) paired with a set of seven distinct RD SET™ DCI® finger sensors.

The number of tested finger sensors was chosen to provide a preliminary insight on performance variability within a pulse oximetry system.

### Testing equipment

In pulse oximetry, simulators work by presenting pulse oximetry systems with optical signals appropriately designed to mimic the way in which the LED light emitted by a finger sensor is modulated when passing through real tissues before reaching the corresponding photodetector.

In this in silico study, we assessed how translucency, perfusion, peripheral oxygen saturation and heart rate (HR) impact on error in the SpO_2_ readings displayed on the tested systems.

We designed a set of test parameters (detailed in Table 1) encompassing, for each of the four variables of interest, a range of values aligned to those expected in the NICU population. We were specifically interested in simulating the most critical values that could be encountered in NICU patients, to challenge pulse oximetry systems performance in conditions not typically assessed in clinical verification studies under current regulatory requirements.

**Table 1:** Simulated parameters and rationale for selection.

Simulated parameters [unit]	Tested values	Rationale
Percent transmission (%T) [%]	0.39 0.87 1.98 4.49	The range of %T values in the NICU studies was 0.43% to 37.70%, with a median value of 3.69% (see Supplementary Materials; %T values were calculated using Eq. (1) and the resulting distribution is shown in Fig. S2). The simulated %T values were chosen to roughly logarithmically span the lower half of the NICU studies data. In addition, the lower end of the NICU %T range was exceeded to account for low representation of subjects with darkly pigmented skin in the NICU studies.
Perfusion (%MOD) [%]	0.2 0.4 0.6 1.1 2.0	The range of %MOD values in the NICU studies was 0.23% to 8.04%, with a median value of 1.30% and with over 90% of the NICU data showing %MOD ≤ 2.0% (see Supplementary Materials; %MOD values were calculated using Eq. (2) and the resulting distribution is shown in Fig. S4). The simulated %MOD parameters were selected to roughly logarithmically span the data range from the NICU studies, slightly exceeding the lower end of the %MOD distribution range.
SpO_2_ [%]	65 70 75 80 85 90 95	The range of simulated SpO_2_ values was selected to roughly span the 5th to 97th percentile of a large population of 468 term and pre-term neonates 5 min after birth [38].
Heart rate (HR) [beats per minute (bpm)]	80 100 120 130	The range of simulated HR values was defined to span the lower end of values that would be expected to be observed in the NICU [22].

**Fig. 1:**
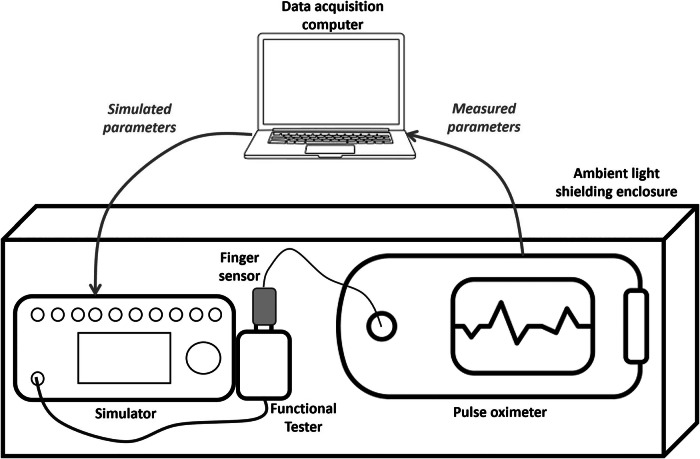
Testing system setup. Simulated parameters, selected based on real-world NICU data, were sent to the pulse oximetry system under test (pulse oximeter + connected finger sensor) via the Simulator and the SpO_2_ Functional Tester. A shielding enclosure ensured all testing equipment was protected from ambient light, which may cause signal artifacts. All parameters measured by the pulse oximetry system under test were then transferred to the data acquisition computer and stored for analysis.

Test parameters were generated using a ProSim™ 8 Vital Signs Simulator (Fluke®, Everett, WA). A SPOT Light SpO_2_ Functional Tester (Fluke®, Everett, WA), consisting of an artificial finger on which the tested finger sensors were applied, was connected to the Simulator. Both Nellcor™ and Masimo® tested finger sensors had their calibration information encoded into the Simulator. Figure 1 provides an overview of the test setup.

### Simulated clinical parameters

#### Translucency and perfusion parameters

Translucency and perfusion were the primary variables of interest for this testing.

Translucency represents the property of materials of allowing light to pass through them. In this work it was expressed in terms of % transmission (%T), a parameter approximately representing the percent amount of a sensor’s LED light that reaches the corresponding photodetector after passing through tissue (see Eq. (1) in the Supplementary Materials for further details). In a clinical setting, %T values depend on both tissue thickness and amount of melanin at monitoring sites, with lower %T typically associated to thick and darkly pigmented sites.

Perfusion is a variable representing the amount of blood in tissues, which varies in a pulsatile fashion. In this work, perfusion was defined in terms of % modulation (%MOD), an indicator of pulsatile optical signal strength that relies on correlation between the amount of light able to pass through tissues and hemodynamic changes [19–21].

Specifically, %MOD represents the percent ratio between the variable (due to attenuation of pulsing blood) and the constant (due to attenuation of other elements, like venous blood, water, bone, and melanin) components of the signal generated by a finger sensor’s LED light passing through a perfused tissue (see Eq. (2) in the Supplementary Materials for further details).

Simulated %T and %MOD parameters (Table 1) were selected based on the distribution of real-world data collected from NICU patients in two prior multicenter observational clinical studies. These studies were designed to follow FDA guidance [15] on verifying safe form, fit, and function of the OxySoft™ neonatal-adult SpO_2_ sensor (Medtronic, Minneapolis, MN) against reference CO-oximetry measurements of arterial oxygen saturation from convenience arterial lines. NICU data were collected using a Nellcor™ OxiMax N-600x patient monitor, equivalent to the one tested in the present work, paired to OxySoft™ neonatal-adult SpO_2_ sensors positioned on the neonates’ feet. Skin tone data were also collected from all NICU patients and categorized using a four-level scale.

These studies were conducted in accordance with the Declaration of Helsinki and all local regulatory requirements and were approved by the Western Institutional Review Board (IRB00000533) and the Timpanogos Regional Hospital’s Institutional Review Board (IRB00003926). Written informed consent, including consent to secondary use of clinical data for research purposes, was obtained from the subjects’ parents. All methods were performed in accordance with applicable governmental and institutional guidelines and regulations. The Supplementary Materials provide additional details on the equations and methods used to calculate %T and %MOD values from the reference NICU datasets and thus derive the set of simulated physiological parameters for testing.

#### SpO_2_ and heart rate parameters

Simulated SpO_2_ and HR parameters were selected based on supporting literature findings, as reported in Table 1. Of note, HR values were specifically selected to reproduce critical real-world conditions known to be challenging for pulse oximetry systems, such as cases of HR < 100 bpm, that usually prompt positive-pressure ventilation or other emergent interventions [22]. For all test cases the Simulator cardiac waveform was set to “Child Normal Sinus Rhythm” (NSR—Pediatric).

### Testing procedure

For both Nellcor™ and Masimo® systems, all seven tested finger sensors were sequentially placed on the SpO_2_ Functional Tester, as the Simulator was cycled automatically through all the combinations of the parameters in Table 1. All simulated values were within the performance range of the Simulator [23]. Parameter variation was performed via a pre-programmed set of nested loops: %T varied most slowly, then %MOD, then SpO_2_, with HR varying in the innermost loop.

Pulse oximetry systems performance was tested in a best-case scenario, with the Simulator respiration and ambient-light artifacts kept inactivated and the system positioned in a light-shielded box to exclude any ambient-light artifact (Fig. 1). For each combination of parameters, data were collected from each finger sensor for 45 s. There were no predefined exclusion criteria for the collected data. Error-handling techniques were considered based on analysis of the collected data. Throughout all test runs, a fiducial mark was used to ensure finger sensors were consistently placed in the same position and orientation with respect to the SpO_2_ Functional Tester.

### Performance metric

The mean SpO_2_ error (expressed as absolute value) was the performance metric for this test. For each combination of Simulator parameters, the mean SpO_2_ error for each system was calculated as the weighted average of the difference over all seven finger sensors between SpO_2_ values reported by the pulse oximetry systems and SpO_2_ values input from the Simulator (Eq. (3) in the Supplementary Materials).

A mean SpO_2_ error of 3% was selected as a threshold to identify potential performance concerns.

### Statistical analysis of SpO_2_ error

An additional analysis was conducted to support a quantitative comparison between Nellcor™ and Masimo® systems across selected points of interest of a signal space defined by the primary variables (%T, %MOD).

First, the mean SpO_2_ error over a defined point of the (%T, %MOD) signal space was calculated for each individual finger sensor (Eq. (4) in the Supplementary Materials).

Then, for each test point, the seven estimates of the mean SpO_2_ error for the Nellcor™ system were compared to the same seven estimates for the Masimo® system using a *t*-test (allowing for unequal variances for the pulse oximeter type).

For this test, we did assume a normal distribution of errors across all test parameters for both systems, as well as a normal distribution of performance amongst the seven tested sensors.

Statistical analyses were run through the *statsmodels* Python package [24]. Statistical significance was defined as *p* < 0.05 at 95% confidence level.

## Results

**Fig. 2:**
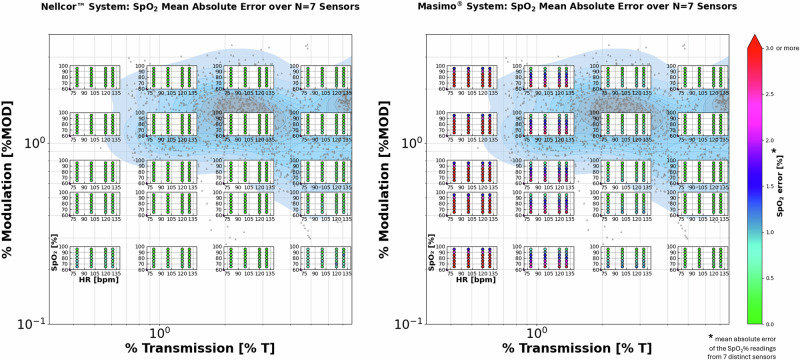
Mean SpO_2_ errors of Nellcor™ and Masimo® pulse oximetry systems as a function of %T (main horizontal axis, logarithmic scale) and %MOD (main vertical axis, logarithmic scale) settings. Each inset graph has its lower-left vertex placed at the (%T, %MOD) coordinates corresponding to the settings for that subset of data acquisition. The colored circles in the inset graphs show the mean absolute value of SpO_2_ error as measured across the seven finger sensors at each individual setting of HR (inset graph, horizontal axis) and SpO_2_ (inset graph, vertical axis). Mean SpO_2_ error is color-coded according to the bar legend on the left of the Figure, with all instances exceeding the 3% threshold displayed in red. The distribution of benchmark clinical NICU data has been overlaid on the (%T, %MOD) space. Each datapoint (gray triangle) represents the median %T/%MOD value of a 10-min epoch of collected NICU data, while the blue shaded region represents their probability density. Deeper colors indicate a higher amount of datapoints in a specific signal space region. Periods of time that the sensor was disconnected or removed from the NICU patients were excluded from the analysis.

Figure 2 shows mean SpO_2_ errors for both Nellcor™ and Masimo® systems over the seven tested sensors as a function of the four test parameters. No error-handling techniques were needed on the data acquired from both systems.

Datapoints in red represent the conditions where the mean SpO_2_ error exceeded the pre-defined threshold of 3%. All numerical results are reported in the Supplementary Materials.

The mean SpO_2_ error for the Nellcor™ system was ≤1.1% % across all parameters explored (0.23 ± 0.24% at %T > 1%; 0.17 ± 0.21% at %T ≤ 1%), suggesting consistent accuracy and repeatability even at the lowest translucency and perfusion settings. The Masimo® system performed well at higher translucencies (mean absolute error 0.33 ± 0.33% at %T > 1%); however, at the lower-translucency settings higher error was reported (2.79 ± 1.62% at %T ≤ 1%) as shown by the red datapoints in Fig. 2, particularly at lower SpO_2_ values, but roughly independently of heart rate. The highest reported mean SpO_2_ error was 1.1% for the Nellcor™ system and 6.4% for the Masimo® system.

To understand the challenging nature of the parameters used for this performance comparison, our reference data distribution derived from NICU clinical trials was overlaid to the same (%T, %MOD) signal space where the SpO_2_ error data were plotted. The NICU data were collected from 34 neonates (mean age 3.3 ± 2.3 days) with the following representation of skin tones: “extremely dark hue”: 1/34 (2.94%), “dark olive hue”: 8/34 (23.53%), “olive hue”: 15/34 (44.12%) and “very light hue”: 10/34 (29.41%).

Figure 2 shows that most of the benchmark NICU data fall in a region of the (%T, %MOD) signal space where both systems showed an average SpO_2_ error <1% (%T > 1%); however, considering the limited representation of neonates with darkly pigmented skin in the reference NICU dataset, it can be hypothesized that a wider real-world NICU distribution could further expand to the left of the plot (at %T < 1%), where the difference in SpO_2_ errors between the two pulse oximetry systems was found to be even higher.

A statistical comparison between mean absolute SpO_2_ errors for Nellcor™ and Masimo® systems was performed for regions of the (%T, %MOD) signal space selected based on the challenging nature of their parameters or on amount (or expected amount) of NICU data represented within them.

Results, reported in Table 2, show statistically significantly lower SpO_2_ errors (*p* < 0.005) for the Nellcor™ system across all tested points of the space, with more consistent differences between the two systems at low %T values.

**Table 2:** Comparison between mean absolute SpO_2_ errors for Nellcor™ and Masimo® systems across different regions of the (%T, %MOD) signal space.

%T [%]	%MOD [%]	Mean absolute SpO_2_ error over all 7 sensors [%]		*t*-test on difference between mean absolute SpO_2_ errors from individual sensors	
		Nellcor™	Masimo®	*p*	Confidence interval (*α* = 0.05)
0.39	0.2	0.55	3.81	0.003	[1.59, 4.93]
0.39	1.1	0.09	3.90	0.001	[2.13, 5.48]
0.87	1.1	0.04	1.70	0.002	[0.91, 2.40]
1.98	1.1	0.01	0.42	0.003	[0.21, 0.63]

Confidence intervals show relatively small variance of the difference between mean absolute SpO_2_ errors, particularly at higher %T and %MOD values.

## Discussion

Since the first introduction of pulse oximeters to the NICU in the 1980s, they have become part of the standard of care and SpO_2_ is widely considered the “fifth vital sign” [25, 26]. Major professional bodies, including the American Heart Association and the American Academy of Pediatrics, have endorsed the central role of continuous pulse oximetry monitoring in targeted oxygen saturation management during neonatal life support and in guiding judicious use of oxygen therapy during neonatal resuscitation, to avoid major morbidities that have been widely shown to be related to hypoxemia or hyperoxemia [2]. In agreement, consensus European guidelines recommend the use of pulse oximetry to monitor infants’ saturation during the first minutes after birth and to adjust oxygenation levels in neonates with respiratory distress syndrome [27].

Pulse oximetry is used throughout NICU hospitalization to manage many aspects of care including the titration of respiratory support and discharge readiness determination [1]. More recently, pulse oximetry has been used for the screening of CCHD in asymptomatic newborns [3, 4] and has demonstrated higher sensitivity compared to alternative strategies (such as prenatal screening and clinical examination) and a low false-positive rate [5].

In each of these use cases, the accuracy of pulse oximetry is vital; failure to intervene in a timely and appropriate manner could impact morbidity and mortality outcomes.

Although all currently available medical-grade pulse oximetry systems must comply with the same regulatory requirements, their real-world performance can vary significantly in conditions that are not typically tested in standardized clinical verification studies but which represent the most challenging portions (in terms of translucency and perfusion) of real-world NICU data distributions. Unfortunately, these challenging conditions are those in which pulse oximetry systems are relied upon most heavily in the clinical practice, as they are representative of instances of significant pathophysiology, such as critically ill neonates with poor perfusion. Although there has been increasing recognition of the limitations of pulse oximetry in recent years [28] (including for neonates, specifically [29, 30]), the overall awareness among health care providers of these potential technical deficiencies and of their impact on the direction or expediency of clinical intervention is still lacking.

In the last few decades, patient simulators have increasingly been used for supplemental bench testing of pulse oximetry system performance; however, as most of this work is done through manufacturer’s pre-market testing or routine inspection in hospital clinical engineering departments, published data is still scarce. Ganesh Kumar et al. recently tested SpO_2_ and pulse rate accuracy of six pulse oximeters using an SpO_2_ simulator and observed performance deterioration in over half of the tested devices in the presence of motion artifact and low perfusion conditions [18]. Although their study did not utilize neonate-specific parameters, their testing method parallels the one used in our study. The authors highlighted the ability of simulators to span a much wider range of variables and to achieve higher repeatability compared to what can typically be done in tests on volunteers in a simulated clinical setting. Further, they encouraged the use of simulators to challenge pulse oximeter performance in physiological or pathological conditions representative of specific patient populations. It is worth remembering, however, that while patient simulators can highlight areas of performance concern that would likely be confirmed in a clinical setting, they are not able to reproduce every facet of the complex physiological behaviors that occur in real subjects and other confounding factors.

In this work, we used a vital sign simulator coupled with a functional tester to investigate differences in the response of two pulse oximetry systems in non-clinically verified conditions representative of NICU patients. Over the entire set of test parameters, both Nellcor™ and Masimo® systems were found to perform within the combined accuracy specifications of each of the oximeters and of the Simulator [23, 31, 32], thus meeting performance requirements defined by current regulatory standards. However, when focusing on low translucency conditions (representative, per Fig. 2, of a consistent portion of a real-world NICU distribution), we observed that the two systems started to deviate in performance, with the Masimo® system reporting mean SpO_2_ errors up to 3.9% in specific areas of interest (Table 2) and up to 6.4% when looking at specific simulated low saturation conditions (Fig. 2 and Supplementary Materials).

Though current standards for pulse oximeters performance verification define a 4% threshold for SpO_2_ accuracy of these equipment when tested on healthy adults [15, 16], we set our threshold for mean SpO_2_ error to 3% considering that stricter requirements are currently being envisioned [33] due to a concern for pulse oximeters disparate performance in the clinical practice [28].

While recognizing that synthetic data can only partially reproduce the variability of a clinical setting, we believe the amount of red datapoints in Fig. 2 justifies the concern that, in critical conditions, some pulse oximeters may show inaccurate readings that, in turn, may lead to erroneous clinical decisions.

Based on recently updated recommendations on CCHD screening in newborns [34], a difference greater than 3% in SpO_2_ readings from two monitoring sites at SpO_2_ levels between 90% and 95% represents a condition for screen failure, and thus prompts additional clinical assessment.

Though the tested oximetry systems didn’t show mean SpO_2_ errors greater than 3% at SpO_2_ levels above 90%, we believe these new recommendations support our selection of a 3% mean SpO_2_ error as a suitable threshold to identify potential performance issues, as the magnitude of reported error shouldn’t exceed the quantity under investigation.

Our findings are further supported by results from a recent study by Gudelunas et al. on healthy adult volunteers with varying skin pigmentation, which also identified performance differences between Nellcor™ and Masimo® pulse oximetry systems [35]. The authors found that SpO_2_ error (defined in their study as the difference between SpO_2_ and oxygen saturation of arterial blood (SaO_2_)) for both devices was dependent on the combined effect of the amount of melanin, perfusion, and degree of hypoxemia. Specifically, they observed that in subjects with darkly pigmented skin and low perfusion, missed hypoxemia events (defined as SaO_2_ values < 88% and corresponding SpO_2_ values between 92% and 96%) were found in 30.2% of the Masimo® readings and in 7.9% of the Nellcor™ readings.

Interestingly, the hypoxia testing by Gudelunas et al. showed a dependence of SpO_2_ error on perfusion, whereas no correlation between SpO_2_ error and %MOD was observed in our testing with the simulator. This finding might be explained by the intrinsic nature of the simulation testing, that allows a satisfactory evaluation of the electro-optical response of pulse oximetry systems to changes in translucency but, as mentioned, is not able to adequately reproduce the physiological tissue-related variables at varying perfusion conditions, which also impacts the response and overall accuracy of this equipment.

Considering that low perfusion states are even more frequent and consequential in real-world NICU populations (as shown by the consistent amount of NICU data at %MOD < 1% in Fig. 2) and the role of pulse oximetry systems in the decision to apply oxygen supplementation in such cases, it should be noted again the importance of not relying solely upon simulator testing for equipment performance characterization. Further research on the optical properties of physiological tissues and their optical interactions with pulse oximetry probes may lead to the development of improved simulators able to adequately reproduce a comprehensive range of real-world subjects and is therefore highly encouraged.

This study was designed to pave the way for the development of new pre-clinical testing methods aimed to obviate some of the limitations of current verification testing required for regulatory approval of pulse oximeters. Due to its pilot nature, it presents some limitations. First, performance was compared in a best-case scenario; a simple heart rate rhythm was set, ambient light was excluded, and no respiratory or other artifacts were activated on the simulator. Second, the dimension of our reference real-world dataset was limited, and so was the representation of darkly pigmented subjects. In addition, skin pigmentation was not assessed using a standardized color scale or spectrophotometric measures (whose usefulness has been highlighted in several recent works [36, 37]), which would be beneficial to include in future studies. Further, as our reference data were derived from NICU patients, the varying contributions of skin thickness and melanin to translucency values during development, or low perfusion conditions typical of the delivery room (which could also impact the width of real-world neonatal data distribution, along both the %T and the %MOD axes) could not be accounted for. However, we expect future, larger, real-world datasets used to inform in silico testing will include greater diversity across more dimensions than those included in our reference sample. It is important to recall that current verification testing conducted per FDA and ISO requirements do not incorporate neonates at all. Third, the performance metric we used (mean SpO_2_ error), which was chosen for the sake of simplicity, is not the one defined in regulatory standards to determine pulse oximeter accuracy. However, as SpO_2_ accuracy is derived from both mean SpO_2_ error and its standard deviation, an increase in mean SpO_2_ error will also impact more complex performance metrics. Fourth, the sensors used in this bench testing (Nellcor™ DS100A-1 and Masimo® RD SET™ DCI® sensors) are not indicated for use on neonates but were chosen due to their compatibility with the selected simulator. Neonate-specific sensors may have different performance characteristics which, however, would not be expected to invalidate the main findings of this work.

Despite these limitations, our study highlights the role that in silico testing can have in pulse oximeters verification. Specifically, this work shows how simulators can be used to extend the range of tested clinical scenarios to include challenging edge cases where pulse oximeter accuracy is most challenged but also most critical in clinical decision making. Although pulse oximeters should not be used as the sole basis for diagnosis or treatment decisions, it remains intuitive that best care is achieved by having the most accurate measurements. Reliability demonstrated through expanded testing, including all clinical conditions of interest, could have a significant impact on both healthcare equity and costs.

## Conclusion

Clinicians should be aware that pulse oximetry system performance may vary based on translucency and perfusion. This laboratory-based work compared SpO_2_ error between two commercially available pulse oximetry systems and identified a persistent performance difference between the two systems at low translucency values, which closely model the characteristics of real NICU patients.

While the current standard of clinical hypoxia testing for regulatory approval of oximeters may be sufficient to assess and verify device performance on many patients in many scenarios, such testing is not comprehensive and does not challenge the oximeters in the settings in which accuracy is most important. Further research in the use of synthetic data for bench testing in pulse oximetry is encouraged. Clinicians, manufacturers and regulators should consider the development of a standardized simulator-based pre-clinical testing method that spans a wide set of perfusion, absorption, and cardiovascular parameters aligned with real-world variability and not systematically reproducible in clinical settings. Such a joint effort would contribute towards more transparent and equitable healthcare and more dependable devices.

## Supplementary information

Supplemental material

## Data Availability

All data that support the findings of this study are included in this article and in the Supplementary Materials. The code used to generate results is available from the corresponding author upon reasonable request.
