# Supplementary material for: Identifying performance differences between two pulse oximetry systems in simulated critical neonatal conditions
Source: J Perinatol. 2025 Jul 30;45(11):1608–14. doi: 10.1038/s41372-025-02364-4 (PMC12660133; doi:10.1038/s41372-025-02364-4)
Supplement: Supplementary file 1 — Supplemental material [file 41372_2025_2364_MOESM1_ESM.pdf]

<u>Supplementary Materials</u>

Identifying Performance Differences Between Two Pulse Oximetry Systems in Simulated Critical Neonatal Conditions

B. King, J. Dove, S. McGonigle, W. Ames, Z. Vesoulis

Table of Contents

1. Configuration of pulse oximetry systems under test..2
2. Testing parameters..2
   2.1. Translucency..2
      2.1.1. Measurement of photocurrent without attenuation...2
      2.1.2. Distribution of translucency data in the benchmark NICU dataset 3
      2.1.3. Characterization of translucency settings on the Simulator...............................4
   2.2. Perfusion 6
      2.2.1. Distribution of perfusion data in the benchmark NICU dataset.........................6
3. Performance Verification...7
   3.1. Determination of SpO_2 error over a defined region of the (%T, %MOD) space.......7
   3.2. Detailed SpO_2 error results 7

1. Configuration of pulse oximetry systems under test

A. Nellcor™ system: a Nellcor™ OxiMax™ N-600x patient monitor (Medtronic, Minneapolis, MN) version 1.7.0.1, configured for normal response mode (factory default) and coupled with a Doc10 cable and a set of seven distinct DS100A-1 finger sensors.

B. Masimo® system: a Rad-97™ Pulse CO-Oximeter® (Masimo®, Irvine, CA; system software version: 1.4.6.2-ss, board 1068, technology level 7e94) configured for continuous monitoring (default mode) and paired with a RD Rainbow SET™ M20-05 cable and a set of seven distinct RD SET™ DCI® finger sensors.

2. Testing parameters

2.1. Translucency

Translucency was expressed as % transmission (%T), calculated as the percent ratio between the photocurrent generated by a sensor's infrared (IR) LED light reaching the corresponding photodetector after passing through tissue (real or simulated) and the same photocurrent reaching the photodetector without any attenuation (Equation 1).

$$\%\mathrm{T} = \frac{\text{Photocurrent through tissue}}{\text{Photocurrent without attenuation}} \times 100$$

Equation 1

Of note, Equation 1 does not account for optical losses due to scattering of the LED light, that in real-world conditions would cause a decrease of the amount of LED light reaching the photodetector. However, this method allows a straightforward measurement of the maximum photocurrent reaching the photodetector without any attenuation (see 2.1.1).

2.1.1. Measurement of photocurrent without attenuation

Photocurrent without attenuation was empirically measured in the lab by applying the Oxysoft™ neonatal-adult SpO_2 sensor, used to collect the NICU data, to the SpO_2 Functional Tester artificial finger upon removal of its internal electronics ("blank finger", Figure 1S). A series of "blank" acquisitions was thus collected, and their average value was used as the denominator of Equation 1 to obtain %T values for all NICU studies data.

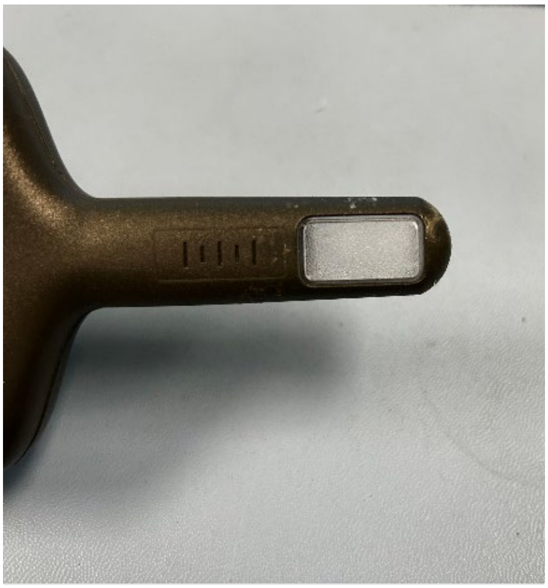

Figure 1S – "Blank finger". The artificial finger of the Fluke® SpO_2 Functional Tester with electronics removed and only the plastic diffusers in between the LED and the photodetector. This was used to simulate a photocurrent path without attenuation.

2.1.2. Distribution of translucency data in the benchmark NICU dataset

Equation 1 was used to calculate %T values from our benchmark NICU dataset. Photocurrent induced by LED light transmitted through tissue was known for all data collected in the NICU studies. Photocurrent without attenuation was estimated using the method presented in 2.1.1 above.

Of note, using Equation 1 to express all translucency data in terms of %T allowed to discard the impact of any LED brightness differences between the different finger sensors used to collect the two sets of data (Nellcor™ OxySoft™ neonatal-adult SpO_2 sensors in the NICU and Nellcor™ DS100A-1 / Masimo® RD SET™ DCI® sensors in the bench testing).

The following Figure 2S represents the distribution of %T data in our reference NICU dataset. %T values marked as test points are those listed in manuscript Table 1 and were used for performance comparison testing between the Nellcor™ and the Masimo® systems.

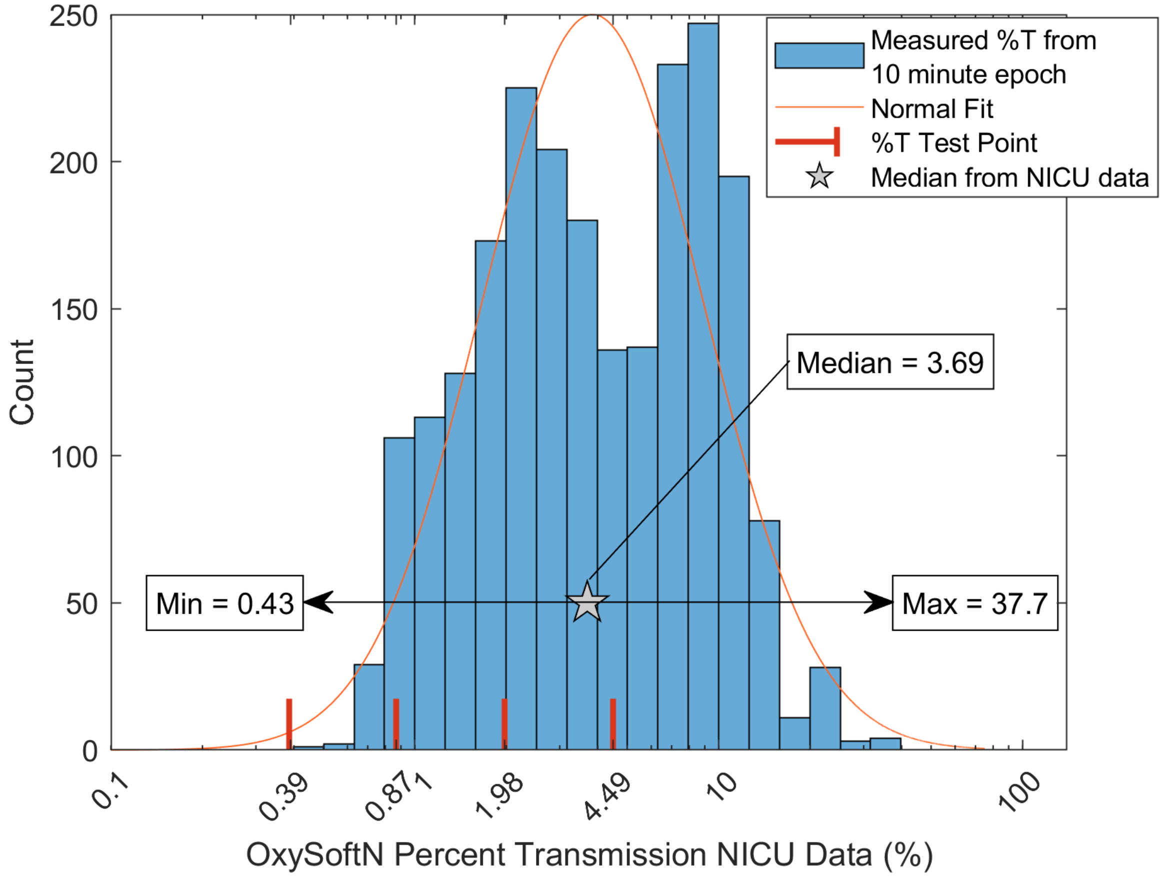

Figure 2S – Distribution of %T data in the reference NICU dataset. Each column in the histogram represents the number of times a defined median % T value was measured in 10-minutes epochs in the dataset.

2.1.3. Characterization of translucency settings on the Simulator

Translucency is expressed by the Fluke® Pro Sim 8 Simulator in parts per million (PPM) units. In order to determine the Simulator PPM values corresponding to the selected test points in Figure 2S above, a characterization of the Simulator in the range 1 - 300 PPM was performed using the Nellcor™ N-600x patient monitor and a DS100A-1 finger sensor (Nellcor™ system per section 1.A above).

Nellcor™ pulse oximeters are designed to adapt both the LED drive current and the gain factor to optimize digitization of the detected signal. However, for the purpose of this characterization, the LED drive current was fixed to 10 mA and the gain factors were held constant.

As done with the OxySoft™ neonatal-adult SpO_2 sensor in 2.1.1, the denominator value for Equation 1 was obtained by averaging photocurrents from a set of acquisitions from the DS100A-1 sensor applied to the "blank finger".

Subsequently, the electronics of the "blank finger" were restored in place, and the DS100A-1 sensor was re-applied to the SpO_2 Functional Tester artificial finger. The Simulator PPM values

were ramped from 1 to 300 PPM (Figure 3S) and the corresponding photocurrent values were recorded from the DS100A-1 sensor, then converted to %T by dividing by the mean photocurrent without attenuation previously calculated.

The Simulator characterization led to the determination of a PPM - %T conversion factor (slope of the fitted line in Figure 3S).

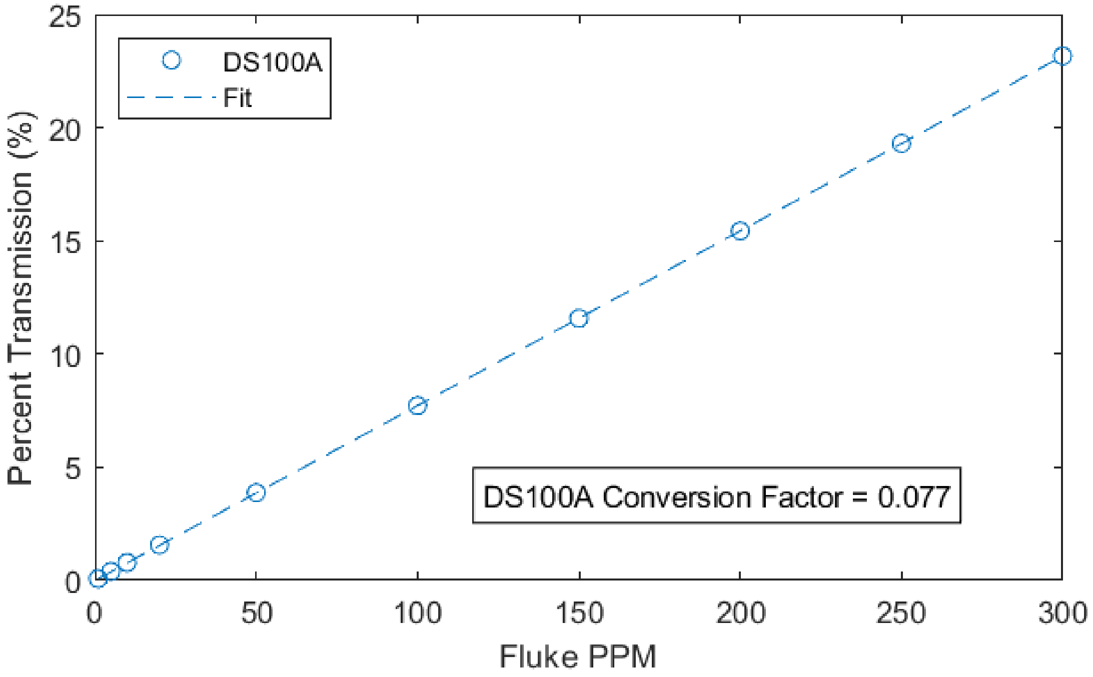

Figure 3S - Characterization of the Fluke® ProSim™ 8 Patient Simulator using a DS100A-1 sensor. Characterization was performed to identify the conversion factor between translucency settings on the Simulator (expressed in PPM units) and percent transmission (%T) values. Data measured in the characterization are shown as open circles and the dashed line is a linear fit (%T = m * PPM, $R^2 = 1$) to the measured data; the fitted slope (m) is shown in the text box as the conversion factor.

2.2. Perfusion

Perfusion was defined as % modulation (%MOD), that is, the ratio between the peak-to-peak (AC) and the mean amplitude (DC) of the photocurrent generated by the IR LED light reaching the corresponding photodetector after passing through an arterial blood flow (Equation 2).

$$\%\text{MOD} = \frac{AC_{IR}}{DC_{IR}} \times 100$$

Equation 2

2.2.1. Distribution of perfusion data in the benchmark NICU dataset

The following Figure 4S represents the distribution of %MOD values (calculated using Equation 2) in our reference NICU dataset. %MOD values marked as test points are those listed in Table 1 in the manuscript and were used for performance comparison testing between the Nellcor™ and the Masimo® systems.

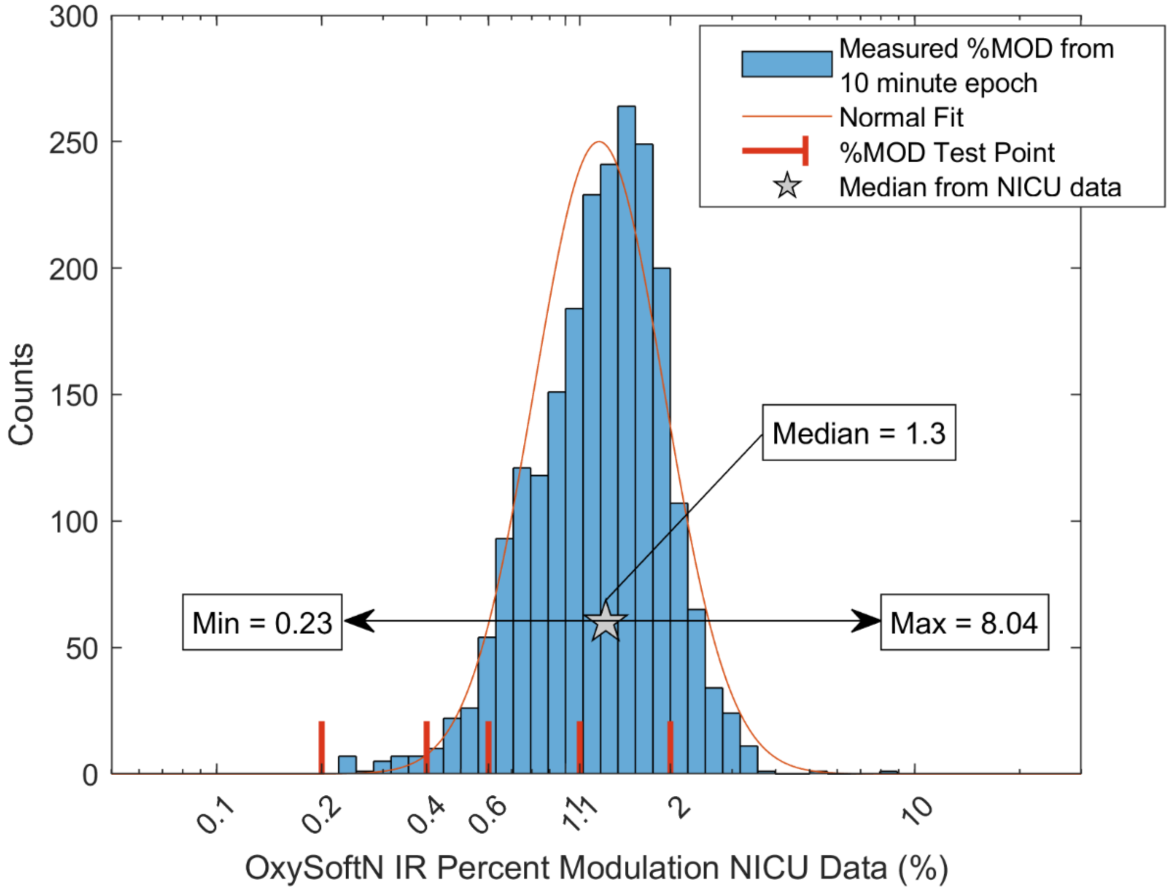

Figure 4S – Distribution of %MOD data in the reference NICU dataset. Each column in the histogram represents the number of times a defined median % MOD value was measured in 10-minutes epochs in the dataset.

3. Performance Verification

At each set of Simulator parameters, the mean SpO_2 error over all seven finger sensors of each type was calculated according to Equation 3, in which $SpO_{2,\,avg}$ was the average SpO_2 reading calculated over the last 10 data points for each set of simulated parameters, and $SpO_{2,\,setting}$ was the SpO_2 value set on the Simulator. The absolute value was considered as it was assumed SpO_2 errors of either sign are undesirable.

$$\Delta SpO_{2,\,mean} = \sum_{i=1}^{7} \frac{|SpO2,avg - SpO2,setting|}{7}$$

Equation 3

As detailed in the Methods, data were acquired for 45 seconds at each set of (HR, SpO_2, %T, %MOD) parameters. Due to the Nellcor and Masimo systems having different reporting frequencies (0.5 and 1 Hz respectively), the number of SpO_2 readings collected from the Masimo system were twice as those collected from the Nellcor system.

To allow both pulse oximetry systems to provide stable readings after settling to every new set of parameters and to compare the two systems based on a same number of data points, only the last ten samples at a given parameters combination were used in calculating $SpO_{2,\,avg}$.

3.1. Determination of SpO_2 error over a defined region of the (%T, %MOD) space

The mean SpO_2 error over a specific region of interest ([%T range], [%MOD range]) was calculated for each individual finger sensor per Equation 4, in which i (ranging from 1 to 7) indicated the tested finger sensor, and N the total number of (HR, SpO_2, %T, %MOD) settings in the evaluated portion of the space.

$$\Delta SpO_{2,\,mean,\,i} = \frac{\sum |SpO2,avg - SpO2,setting|}{N}$$

Equation 4

3.2. Detailed SpO_2 error results

Table 1S reports SpO_2 errors measured on both systems under test at each combination of the test parameters. These data align to those presented in Figure 2 of the manuscript.

Table 1S – Measured SpO_2 errors at each test parameters combination

Translucency (%)	Perfusion (%)	SpO_2 (%)	HR (bpm)	SpO_2 error – Nellcor (%)	SpO_2 error – Masimo (%)
				For each system under test, SpO_2 error is presented as the mean absolute error of the SpO_2% readings from 7 distinct sensors	
0.39	0.2	95	130	0.50	1.62
0.39	0.2	95	80	0.23	1.78
0.39	0.2	95	100	0.17	1.64
0.39	0.2	95	120	0.37	1.52
0.39	0.2	65	130	0.87	6.04
0.39	0.2	65	80	1.01	6.16
0.39	0.2	65	100	0.56	6.38
0.39	0.2	65	120	0.53	6.07
0.39	0.2	70	130	0.59	5.04
0.39	0.2	70	80	0.90	4.78
0.39	0.2	70	100	0.49	5.11
0.39	0.2	70	120	0.67	5.05
0.39	0.2	75	130	0.44	4.50
0.39	0.2	75	80	0.93	4.50
0.39	0.2	75	100	0.50	4.26
0.39	0.2	75	120	0.67	4.63
0.39	0.2	80	130	0.91	3.89
0.39	0.2	80	80	0.67	4.09
0.39	0.2	80	100	0.50	4.16
0.39	0.2	80	120	0.80	3.93
0.39	0.2	85	130	0.33	3.01
0.39	0.2	85	80	0.70	3.30
0.39	0.2	85	100	0.50	3.13
0.39	0.2	85	120	0.54	3.07
0.39	0.2	90	130	0.31	2.22
0.39	0.2	90	80	0.26	2.37
0.39	0.2	90	100	0.21	2.33
0.39	0.2	90	120	0.23	2.20
0.39	0.4	95	130	0.01	1.54
0.39	0.4	95	80	0.09	1.61
0.39	0.4	95	100	0.00	1.59
0.39	0.4	95	120	0.03	1.50
0.39	0.4	65	130	0.39	6.23
0.39	0.4	65	80	0.49	5.80
0.39	0.4	65	100	0.56	6.04
0.39	0.4	65	120	0.43	6.14
0.39	0.4	70	130	0.39	5.51
0.39	0.4	70	80	0.17	5.24

Translucency (%)	Perfusion (%)	SpO_2 (%)	HR (bpm)	SpO_2 error – Nellcor (%)	SpO_2 error – Masimo (%)
				For each system under test, SpO_2 error is presented as the mean absolute error of the SpO_2% readings from 7 distinct sensors	
0.39	0.4	70	100	0.33	5.37
0.39	0.4	70	120	0.63	5.50
0.39	0.4	75	130	0.47	4.42
0.39	0.4	75	80	0.31	4.74
0.39	0.4	75	100	0.29	4.52
0.39	0.4	75	120	0.46	4.67
0.39	0.4	80	130	0.33	3.84
0.39	0.4	80	80	0.54	3.79
0.39	0.4	80	100	0.23	3.85
0.39	0.4	80	120	0.27	3.88
0.39	0.4	85	130	0.13	3.05
0.39	0.4	85	80	0.16	3.22
0.39	0.4	85	100	0.06	3.03
0.39	0.4	85	120	0.19	3.14
0.39	0.4	90	130	0.13	2.37
0.39	0.4	90	80	0.11	2.37
0.39	0.4	90	100	0.11	2.33
0.39	0.4	90	120	0.33	2.35
0.39	0.6	95	130	0.00	1.61
0.39	0.6	95	80	0.00	1.57
0.39	0.6	95	100	0.06	1.52
0.39	0.6	95	120	0.04	1.54
0.39	0.6	65	130	0.40	6.17
0.39	0.6	65	80	0.50	6.09
0.39	0.6	65	100	0.39	6.27
0.39	0.6	65	120	0.36	6.16
0.39	0.6	70	130	0.41	5.40
0.39	0.6	70	80	0.30	5.35
0.39	0.6	70	100	0.47	5.39
0.39	0.6	70	120	0.29	5.45
0.39	0.6	75	130	0.26	4.78
0.39	0.6	75	80	0.36	4.67
0.39	0.6	75	100	0.31	4.66
0.39	0.6	75	120	0.24	4.69
0.39	0.6	80	130	0.19	3.89
0.39	0.6	80	80	0.27	3.94
0.39	0.6	80	100	0.14	3.85
0.39	0.6	80	120	0.26	3.86
0.39	0.6	85	130	0.04	3.09

Translucency (%)	Perfusion (%)	SpO_2 (%)	HR (bpm)	SpO_2 error – Nellcor (%)	SpO_2 error – Masimo (%)
				For each system under test, SpO_2 error is presented as the mean absolute error of the SpO_2% readings from 7 distinct sensors	
0.39	0.6	85	80	0.17	3.00
0.39	0.6	85	100	0.21	3.19
0.39	0.6	85	120	0.20	3.21
0.39	0.6	90	130	0.14	2.46
0.39	0.6	90	80	0.01	2.39
0.39	0.6	90	100	0.11	2.33
0.39	0.6	90	120	0.10	2.33
0.39	1.1	95	130	0.00	1.57
0.39	1.1	95	80	0.00	1.57
0.39	1.1	95	100	0.00	1.57
0.39	1.1	95	120	0.00	1.57
0.39	1.1	65	130	0.24	6.22
0.39	1.1	65	80	0.30	6.15
0.39	1.1	65	100	0.13	6.18
0.39	1.1	65	120	0.17	6.21
0.39	1.1	70	130	0.20	5.43
0.39	1.1	70	80	0.23	5.44
0.39	1.1	70	100	0.20	5.47
0.39	1.1	70	120	0.14	5.44
0.39	1.1	75	130	0.07	4.71
0.39	1.1	75	80	0.16	4.85
0.39	1.1	75	100	0.14	4.66
0.39	1.1	75	120	0.17	4.64
0.39	1.1	80	130	0.01	3.79
0.39	1.1	80	80	0.07	3.76
0.39	1.1	80	100	0.01	3.79
0.39	1.1	80	120	0.20	3.94
0.39	1.1	85	130	0.07	3.01
0.39	1.1	85	80	0.06	3.09
0.39	1.1	85	100	0.03	3.13
0.39	1.1	85	120	0.04	3.03
0.39	1.1	90	130	0.00	2.47
0.39	1.1	90	80	0.00	2.49
0.39	1.1	90	100	0.00	2.51
0.39	1.1	90	120	0.00	2.40
0.39	2	95	130	0.00	1.53
0.39	2	95	80	0.00	1.57
0.39	2	95	100	0.00	1.57
0.39	2	95	120	0.00	1.54

Translucency (%)	Perfusion (%)	SpO_2 (%)	HR (bpm)	SpO_2 error – Nellcor (%)	SpO_2 error – Masimo (%)
				For each system under test, SpO_2 error is presented as the mean absolute error of the SpO_2% readings from 7 distinct sensors	
0.39	2	65	130	0.01	6.44
0.39	2	65	80	0.00	6.31
0.39	2	65	100	0.00	6.38
0.39	2	65	120	0.01	6.30
0.39	2	70	130	0.00	5.59
0.39	2	70	80	0.01	5.45
0.39	2	70	100	0.03	5.64
0.39	2	70	120	0.03	5.66
0.39	2	75	130	0.00	4.84
0.39	2	75	80	0.01	4.86
0.39	2	75	100	0.00	4.79
0.39	2	75	120	0.00	4.76
0.39	2	80	130	0.00	3.84
0.39	2	80	80	0.00	4.00
0.39	2	80	100	0.00	3.91
0.39	2	80	120	0.00	3.91
0.39	2	85	130	0.00	3.07
0.39	2	85	80	0.00	3.07
0.39	2	85	100	0.00	3.09
0.39	2	85	120	0.00	3.09
0.39	2	90	130	0.00	2.47
0.39	2	90	80	0.00	2.46
0.39	2	90	100	0.00	2.47
0.39	2	90	120	0.00	2.40
0.87	0.2	95	130	0.06	0.52
0.87	0.2	95	80	0.10	0.78
0.87	0.2	95	100	0.04	0.76
0.87	0.2	95	120	0.06	0.61
0.87	0.2	65	130	0.49	2.74
0.87	0.2	65	80	0.41	2.46
0.87	0.2	65	100	0.51	2.75
0.87	0.2	65	120	0.44	2.76
0.87	0.2	70	130	0.39	2.34
0.87	0.2	70	80	0.49	2.46
0.87	0.2	70	100	0.49	2.39
0.87	0.2	70	120	0.54	2.46
0.87	0.2	75	130	0.17	2.09
0.87	0.2	75	80	0.39	1.92
0.87	0.2	75	100	0.36	2.04

Translucency (%)	Perfusion (%)	SpO_2 (%)	HR (bpm)	SpO_2 error – Nellcor (%)	SpO_2 error – Masimo (%)
				For each system under test, SpO_2 error is presented as the mean absolute error of the SpO_2% readings from 7 distinct sensors	
0.87	0.2	75	120	0.37	1.97
0.87	0.2	80	130	0.23	1.54
0.87	0.2	80	80	0.23	1.59
0.87	0.2	80	100	0.33	1.67
0.87	0.2	80	120	0.26	1.71
0.87	0.2	85	130	0.14	1.18
0.87	0.2	85	80	0.24	1.41
0.87	0.2	85	100	0.23	1.45
0.87	0.2	85	120	0.14	1.34
0.87	0.2	90	130	0.20	1.08
0.87	0.2	90	80	0.14	1.00
0.87	0.2	90	100	0.10	1.13
0.87	0.2	90	120	0.44	1.03
0.87	0.4	95	130	0.00	0.60
0.87	0.4	95	80	0.03	0.60
0.87	0.4	95	100	0.00	0.60
0.87	0.4	95	120	0.00	0.66
0.87	0.4	65	130	0.36	2.75
0.87	0.4	65	80	0.41	2.69
0.87	0.4	65	100	0.16	2.71
0.87	0.4	65	120	0.33	2.82
0.87	0.4	70	130	0.34	2.38
0.87	0.4	70	80	0.27	2.44
0.87	0.4	70	100	0.19	2.41
0.87	0.4	70	120	0.34	2.48
0.87	0.4	75	130	0.33	2.01
0.87	0.4	75	80	0.31	2.06
0.87	0.4	75	100	0.26	1.96
0.87	0.4	75	120	0.09	1.95
0.87	0.4	80	130	0.06	1.59
0.87	0.4	80	80	0.17	1.64
0.87	0.4	80	100	0.27	1.58
0.87	0.4	80	120	0.19	1.63
0.87	0.4	85	130	0.04	1.50
0.87	0.4	85	80	0.14	1.31
0.87	0.4	85	100	0.11	1.44
0.87	0.4	85	120	0.10	1.36
0.87	0.4	90	130	0.01	1.08
0.87	0.4	90	80	0.03	1.05

Translucency (%)	Perfusion (%)	SpO_2 (%)	HR (bpm)	SpO_2 error – Nellcor (%)	SpO_2 error – Masimo (%)
				For each system under test, SpO_2 error is presented as the mean absolute error of the SpO_2% readings from 7 distinct sensors	
0.87	0.4	90	100	0.03	1.05
0.87	0.4	90	120	0.00	0.99
0.87	0.6	95	130	0.00	0.48
0.87	0.6	95	80	0.00	0.69
0.87	0.6	95	100	0.00	0.49
0.87	0.6	95	120	0.00	0.57
0.87	0.6	65	130	0.23	2.64
0.87	0.6	65	80	0.31	2.71
0.87	0.6	65	100	0.14	2.69
0.87	0.6	65	120	0.19	2.70
0.87	0.6	70	130	0.16	2.55
0.87	0.6	70	80	0.21	2.44
0.87	0.6	70	100	0.19	2.56
0.87	0.6	70	120	0.24	2.52
0.87	0.6	75	130	0.11	2.03
0.87	0.6	75	80	0.31	1.91
0.87	0.6	75	100	0.11	2.00
0.87	0.6	75	120	0.19	2.06
0.87	0.6	80	130	0.09	1.57
0.87	0.6	80	80	0.06	1.69
0.87	0.6	80	100	0.03	1.57
0.87	0.6	80	120	0.04	1.57
0.87	0.6	85	130	0.10	1.46
0.87	0.6	85	80	0.06	1.44
0.87	0.6	85	100	0.10	1.37
0.87	0.6	85	120	0.03	1.41
0.87	0.6	90	130	0.00	1.03
0.87	0.6	90	80	0.00	1.04
0.87	0.6	90	100	0.00	1.06
0.87	0.6	90	120	0.00	1.06
0.87	1.1	95	130	0.00	0.59
0.87	1.1	95	80	0.00	0.49
0.87	1.1	95	100	0.00	0.46
0.87	1.1	95	120	0.00	0.56
0.87	1.1	65	130	0.01	2.71
0.87	1.1	65	80	0.04	2.72
0.87	1.1	65	100	0.07	2.71
0.87	1.1	65	120	0.01	2.72
0.87	1.1	70	130	0.14	2.57

Translucency (%)	Perfusion (%)	SpO_2 (%)	HR (bpm)	SpO_2 error – Nellcor (%)	SpO_2 error – Masimo (%)
				For each system under test, SpO_2 error is presented as the mean absolute error of the SpO_2% readings from 7 distinct sensors	
0.87	1.1	70	80	0.06	2.54
0.87	1.1	70	100	0.11	2.56
0.87	1.1	70	120	0.06	2.57
0.87	1.1	75	130	0.11	2.04
0.87	1.1	75	80	0.11	2.03
0.87	1.1	75	100	0.03	2.00
0.87	1.1	75	120	0.10	2.08
0.87	1.1	80	130	0.03	1.57
0.87	1.1	80	80	0.04	1.62
0.87	1.1	80	100	0.00	1.57
0.87	1.1	80	120	0.14	1.57
0.87	1.1	85	130	0.00	1.36
0.87	1.1	85	80	0.00	1.43
0.87	1.1	85	100	0.01	1.41
0.87	1.1	85	120	0.00	1.40
0.87	1.1	90	130	0.00	1.00
0.87	1.1	90	80	0.00	1.03
0.87	1.1	90	100	0.00	1.09
0.87	1.1	90	120	0.00	1.06
0.87	2.0	95	130	0.00	0.39
0.87	2.0	95	80	0.00	0.38
0.87	2.0	95	100	0.00	0.43
0.87	2.0	95	120	0.00	0.43
0.87	2.0	65	130	0.00	2.78
0.87	2.0	65	80	0.01	2.84
0.87	2.0	65	100	0.00	2.86
0.87	2.0	65	120	0.00	2.86
0.87	2.0	70	130	0.00	2.57
0.87	2.0	70	80	0.00	2.57
0.87	2.0	70	100	0.00	2.57
0.87	2.0	70	120	0.00	2.57
0.87	2.0	75	130	0.00	2.11
0.87	2.0	75	80	0.00	2.20
0.87	2.0	75	100	0.00	2.14
0.87	2.0	75	120	0.00	2.14
0.87	2.0	80	130	0.00	1.57
0.87	2.0	80	80	0.00	1.57
0.87	2.0	80	100	0.00	1.57
0.87	2.0	80	120	0.00	1.57

Translucency (%)	Perfusion (%)	SpO_2 (%)	HR (bpm)	SpO_2 error – Nellcor (%)	SpO_2 error – Masimo (%)
				For each system under test, SpO_2 error is presented as the mean absolute error of the SpO_2% readings from 7 distinct sensors	
0.87	2.0	85	130	0.00	1.39
0.87	2.0	85	80	0.00	1.41
0.87	2.0	85	100	0.00	1.40
0.87	2.0	85	120	0.00	1.39
0.87	2.0	90	130	0.00	1.00
0.87	2.0	90	80	0.00	1.00
0.87	2.0	90	100	0.00	1.00
0.87	2.0	90	120	0.00	1.00
1.98	0.2	95	130	0.00	0.14
1.98	0.2	95	80	0.00	0.14
1.98	0.2	95	100	0.00	0.09
1.98	0.2	95	120	0.00	0.14
1.98	0.2	65	130	0.34	1.03
1.98	0.2	65	80	0.40	1.15
1.98	0.2	65	100	0.40	1.16
1.98	0.2	65	120	0.33	1.19
1.98	0.2	70	130	0.20	1.01
1.98	0.2	70	80	0.37	0.97
1.98	0.2	70	100	0.39	1.01
1.98	0.2	70	120	0.27	0.98
1.98	0.2	75	130	0.33	0.99
1.98	0.2	75	80	0.31	0.96
1.98	0.2	75	100	0.23	1.01
1.98	0.2	75	120	0.41	0.81
1.98	0.2	80	130	0.06	0.66
1.98	0.2	80	80	0.19	0.64
1.98	0.2	80	100	0.21	0.61
1.98	0.2	80	120	0.23	0.73
1.98	0.2	85	130	0.07	0.55
1.98	0.2	85	80	0.07	0.41
1.98	0.2	85	100	0.07	0.49
1.98	0.2	85	120	0.13	0.41
1.98	0.2	90	130	0.04	0.19
1.98	0.2	90	80	0.00	0.19
1.98	0.2	90	100	0.10	0.19
1.98	0.2	90	120	0.07	0.28
1.98	0.4	95	130	0.00	0.11
1.98	0.4	95	80	0.00	0.11
1.98	0.4	95	100	0.00	0.14

Translucency (%)	Perfusion (%)	SpO_2 (%)	HR (bpm)	SpO_2 error – Nellcor (%)	SpO_2 error – Masimo (%)
				For each system under test, SpO_2 error is presented as the mean absolute error of the SpO_2% readings from 7 distinct sensors	
1.98	0.4	95	120	0.00	0.14
1.98	0.4	65	130	0.19	1.05
1.98	0.4	65	80	0.19	1.00
1.98	0.4	65	100	0.19	1.04
1.98	0.4	65	120	0.23	1.00
1.98	0.4	70	130	0.26	0.89
1.98	0.4	70	80	0.21	0.95
1.98	0.4	70	100	0.17	1.00
1.98	0.4	70	120	0.21	0.93
1.98	0.4	75	130	0.17	0.77
1.98	0.4	75	80	0.06	0.86
1.98	0.4	75	100	0.10	0.77
1.98	0.4	75	120	0.13	0.81
1.98	0.4	80	130	0.10	0.53
1.98	0.4	80	80	0.03	0.47
1.98	0.4	80	100	0.06	0.49
1.98	0.4	80	120	0.06	0.51
1.98	0.4	85	130	0.01	0.29
1.98	0.4	85	80	0.00	0.30
1.98	0.4	85	100	0.01	0.34
1.98	0.4	85	120	0.00	0.31
1.98	0.4	90	130	0.00	0.04
1.98	0.4	90	80	0.00	0.06
1.98	0.4	90	100	0.00	0.00
1.98	0.4	90	120	0.00	0.04
1.98	0.6	95	130	0.00	0.00
1.98	0.6	95	80	0.00	0.00
1.98	0.6	95	100	0.00	0.00
1.98	0.6	95	120	0.00	0.00
1.98	0.6	65	130	0.24	0.96
1.98	0.6	65	80	0.27	1.00
1.98	0.6	65	100	0.20	0.94
1.98	0.6	65	120	0.23	0.98
1.98	0.6	70	130	0.23	0.86
1.98	0.6	70	80	0.29	0.82
1.98	0.6	70	100	0.20	0.79
1.98	0.6	70	120	0.17	0.80
1.98	0.6	75	130	0.07	0.60
1.98	0.6	75	80	0.06	0.69

Translucency (%)	Perfusion (%)	SpO_2 (%)	HR (bpm)	SpO_2 error – Nellcor (%)	SpO_2 error – Masimo (%)
				For each system under test, SpO_2 error is presented as the mean absolute error of the SpO_2% readings from 7 distinct sensors	
1.98	0.6	75	100	0.07	0.70
1.98	0.6	75	120	0.14	0.48
1.98	0.6	80	130	0.04	0.38
1.98	0.6	80	80	0.04	0.38
1.98	0.6	80	100	0.03	0.44
1.98	0.6	80	120	0.06	0.43
1.98	0.6	85	130	0.00	0.14
1.98	0.6	85	80	0.00	0.18
1.98	0.6	85	100	0.00	0.14
1.98	0.6	85	120	0.00	0.11
1.98	0.6	90	130	0.00	0.00
1.98	0.6	90	80	0.00	0.00
1.98	0.6	90	100	0.00	0.00
1.98	0.6	90	120	0.00	0.00
1.98	1.1	95	130	0.00	0.00
1.98	1.1	95	80	0.00	0.00
1.98	1.1	95	100	0.00	0.00
1.98	1.1	95	120	0.00	0.00
1.98	1.1	65	130	0.00	0.90
1.98	1.1	65	80	0.06	0.86
1.98	1.1	65	100	0.04	0.86
1.98	1.1	65	120	0.00	0.86
1.98	1.1	70	130	0.00	0.84
1.98	1.1	70	80	0.01	0.83
1.98	1.1	70	100	0.01	0.76
1.98	1.1	70	120	0.01	0.86
1.98	1.1	75	130	0.00	0.64
1.98	1.1	75	80	0.00	0.65
1.98	1.1	75	100	0.01	0.67
1.98	1.1	75	120	0.00	0.66
1.98	1.1	80	130	0.00	0.47
1.98	1.1	80	80	0.00	0.44
1.98	1.1	80	100	0.00	0.47
1.98	1.1	80	120	0.00	0.47
1.98	1.1	85	130	0.00	0.14
1.98	1.1	85	80	0.00	0.16
1.98	1.1	85	100	0.00	0.14
1.98	1.1	85	120	0.00	0.14
1.98	1.1	90	130	0.00	0.00

Translucency (%)	Perfusion (%)	SpO_2 (%)	HR (bpm)	SpO_2 error – Nellcor (%)	SpO_2 error – Masimo (%)
				For each system under test, SpO_2 error is presented as the mean absolute error of the SpO_2% readings from 7 distinct sensors	
1.98	1.1	90	80	0.00	0.02
1.98	1.1	90	100	0.00	0.00
1.98	1.1	90	120	0.00	0.05
1.98	2.0	95	130	0.00	0.00
1.98	2.0	95	80	0.00	0.00
1.98	2.0	95	100	0.00	0.00
1.98	2.0	95	120	0.00	0.00
1.98	2.0	65	130	0.00	1.00
1.98	2.0	65	80	0.00	0.86
1.98	2.0	65	100	0.00	0.89
1.98	2.0	65	120	0.00	0.96
1.98	2.0	70	130	0.00	0.86
1.98	2.0	70	80	0.00	0.89
1.98	2.0	70	100	0.00	0.86
1.98	2.0	70	120	0.00	0.86
1.98	2.0	75	130	0.00	0.78
1.98	2.0	75	80	0.00	0.80
1.98	2.0	75	100	0.00	0.84
1.98	2.0	75	120	0.00	0.79
1.98	2.0	80	130	0.00	0.49
1.98	2.0	80	80	0.00	0.55
1.98	2.0	80	100	0.00	0.54
1.98	2.0	80	120	0.00	0.53
1.98	2.0	85	130	0.00	0.24
1.98	2.0	85	80	0.00	0.22
1.98	2.0	85	100	0.00	0.20
1.98	2.0	85	120	0.00	0.22
1.98	2.0	90	130	0.00	0.00
1.98	2.0	90	80	0.00	0.00
1.98	2.0	90	100	0.00	0.00
1.98	2.0	90	120	0.00	0.04
4.49	0.2	95	130	0.16	0.06
4.49	0.2	95	80	0.83	0.07
4.49	0.2	95	100	0.56	0.00
4.49	0.2	95	120	0.39	0.04
4.49	0.2	65	130	0.67	0.39
4.49	0.2	65	80	0.67	0.52
4.49	0.2	65	100	0.47	0.48
4.49	0.2	65	120	0.83	0.28

Translucency (%)	Perfusion (%)	SpO_2 (%)	HR (bpm)	SpO_2 error – Nellcor (%)	SpO_2 error – Masimo (%)
				For each system under test, SpO_2 error is presented as the mean absolute error of the SpO_2% readings from 7 distinct sensors	
4.49	0.2	70	130	0.83	0.59
4.49	0.2	70	80	0.79	0.14
4.49	0.2	70	100	0.67	0.16
4.49	0.2	70	120	1.14	0.44
4.49	0.2	75	130	0.59	0.19
4.49	0.2	75	80	0.77	0.19
4.49	0.2	75	100	0.94	0.32
4.49	0.2	75	120	0.60	0.43
4.49	0.2	80	130	0.63	0.21
4.49	0.2	80	80	0.74	0.31
4.49	0.2	80	100	0.59	0.33
4.49	0.2	80	120	0.69	0.29
4.49	0.2	85	130	1.13	0.15
4.49	0.2	85	80	0.77	0.26
4.49	0.2	85	100	0.60	0.16
4.49	0.2	85	120	0.41	0.24
4.49	0.2	90	130	0.29	0.06
4.49	0.2	90	80	0.60	0.10
4.49	0.2	90	100	0.29	0.09
4.49	0.2	90	120	0.63	0.14
4.49	0.4	95	130	0.11	0.00
4.49	0.4	95	80	0.23	0.00
4.49	0.4	95	100	0.10	0.00
4.49	0.4	95	120	0.06	0.01
4.49	0.4	65	130	0.59	0.29
4.49	0.4	65	80	0.49	0.32
4.49	0.4	65	100	0.60	0.22
4.49	0.4	65	120	0.49	0.41
4.49	0.4	70	130	0.46	0.28
4.49	0.4	70	80	0.86	0.18
4.49	0.4	70	100	0.53	0.21
4.49	0.4	70	120	0.57	0.20
4.49	0.4	75	130	0.41	0.14
4.49	0.4	75	80	0.83	0.22
4.49	0.4	75	100	0.77	0.12
4.49	0.4	75	120	0.47	0.13
4.49	0.4	80	130	0.43	0.19
4.49	0.4	80	80	0.71	0.14
4.49	0.4	80	100	0.59	0.08

Translucency (%)	Perfusion (%)	SpO_2 (%)	HR (bpm)	SpO_2 error – Nellcor (%)	SpO_2 error – Masimo (%)
				For each system under test, SpO_2 error is presented as the mean absolute error of the SpO_2% readings from 7 distinct sensors	
4.49	0.4	80	120	0.54	0.18
4.49	0.4	85	130	0.33	0.05
4.49	0.4	85	80	0.36	0.08
4.49	0.4	85	100	0.17	0.01
4.49	0.4	85	120	0.13	0.07
4.49	0.4	90	130	0.27	0.00
4.49	0.4	90	80	0.21	0.00
4.49	0.4	90	100	0.11	0.04
4.49	0.4	90	120	0.17	0.01
4.49	0.6	95	130	0.00	0.00
4.49	0.6	95	80	0.16	0.00
4.49	0.6	95	100	0.03	0.00
4.49	0.6	95	120	0.07	0.00
4.49	0.6	65	130	0.20	0.22
4.49	0.6	65	80	0.44	0.39
4.49	0.6	65	100	0.46	0.26
4.49	0.6	65	120	0.54	0.22
4.49	0.6	70	130	0.61	0.24
4.49	0.6	70	80	0.21	0.30
4.49	0.6	70	100	0.50	0.20
4.49	0.6	70	120	0.34	0.23
4.49	0.6	75	130	0.47	0.19
4.49	0.6	75	80	0.54	0.16
4.49	0.6	75	100	0.34	0.19
4.49	0.6	75	120	0.39	0.22
4.49	0.6	80	130	0.29	0.02
4.49	0.6	80	80	0.54	0.15
4.49	0.6	80	100	0.41	0.07
4.49	0.6	80	120	0.39	0.09
4.49	0.6	85	130	0.20	0.01
4.49	0.6	85	80	0.27	0.04
4.49	0.6	85	100	0.34	0.01
4.49	0.6	85	120	0.26	0.03
4.49	0.6	90	130	0.26	0.00
4.49	0.6	90	80	0.24	0.01
4.49	0.6	90	100	0.17	0.00
4.49	0.6	90	120	0.29	0.00
4.49	1.1	95	130	0.11	0.00
4.49	1.1	95	80	0.09	0.06

Translucency (%)	Perfusion (%)	SpO_2 (%)	HR (bpm)	SpO_2 error – Nellcor (%)	SpO_2 error – Masimo (%)
				For each system under test, SpO_2 error is presented as the mean absolute error of the SpO_2% readings from 7 distinct sensors	
4.49	1.1	95	100	0.04	0.00
4.49	1.1	95	120	0.09	0.00
4.49	1.1	65	130	0.54	0.38
4.49	1.1	65	80	0.36	0.39
4.49	1.1	65	100	0.59	0.34
4.49	1.1	65	120	0.34	0.36
4.49	1.1	70	130	0.59	0.21
4.49	1.1	70	80	0.50	0.27
4.49	1.1	70	100	0.59	0.26
4.49	1.1	70	120	0.34	0.21
4.49	1.1	75	130	0.36	0.12
4.49	1.1	75	80	0.47	0.16
4.49	1.1	75	100	0.26	0.21
4.49	1.1	75	120	0.41	0.17
4.49	1.1	80	130	0.23	0.11
4.49	1.1	80	80	0.36	0.19
4.49	1.1	80	100	0.26	0.13
4.49	1.1	80	120	0.27	0.13
4.49	1.1	85	130	0.13	0.11
4.49	1.1	85	80	0.16	0.05
4.49	1.1	85	100	0.19	0.03
4.49	1.1	85	120	0.14	0.08
4.49	1.1	90	130	0.16	0.00
4.49	1.1	90	80	0.10	0.08
4.49	1.1	90	100	0.11	0.00
4.49	1.1	90	120	0.10	0.00
4.49	2.0	95	130	0.03	0.00
4.49	2.0	95	80	0.04	0.00
4.49	2.0	95	100	0.06	0.00
4.49	2.0	95	120	0.04	0.00
4.49	2.0	65	130	0.37	0.43
4.49	2.0	65	80	0.69	0.44
4.49	2.0	65	100	0.53	0.43
4.49	2.0	65	120	0.57	0.45
4.49	2.0	70	130	0.31	0.33
4.49	2.0	70	80	0.39	0.31
4.49	2.0	70	100	0.49	0.24
4.49	2.0	70	120	0.33	0.31
4.49	2.0	75	130	0.34	0.21

Translucency (%)	Perfusion (%)	SpO_2 (%)	HR (bpm)	SpO_2 error – Nellcor (%)	SpO_2 error – Masimo (%)
				For each system under test, SpO_2 error is presented as the mean absolute error of the SpO_2% readings from 7 distinct sensors	
4.49	2.0	75	80	0.34	0.34
4.49	2.0	75	100	0.29	0.24
4.49	2.0	75	120	0.20	0.21
4.49	2.0	80	130	0.19	0.14
4.49	2.0	80	80	0.17	0.14
4.49	2.0	80	100	0.17	0.20
4.49	2.0	80	120	0.17	0.16
4.49	2.0	85	130	0.14	0.06
4.49	2.0	85	80	0.16	0.11
4.49	2.0	85	100	0.14	0.10
4.49	2.0	85	120	0.17	0.06
4.49	2.0	90	130	0.09	0.00
4.49	2.0	90	80	0.13	0.01
4.49	2.0	90	100	0.14	0.00
4.49	2.0	90	120	0.13	0.00
